# Comparative Bioaccesibility Study of Cereal-Based Nutraceutical Ingredients Using INFOGEST Static, Semi-Dynamic and Dynamic In Vitro Gastrointestinal Digestion

**DOI:** 10.3390/antiox13101244

**Published:** 2024-10-16

**Authors:** Iván Jesús Jiménez-Pulido, Ana Belén Martín-Diana, Daniel de Luis, Daniel Rico

**Affiliations:** 1 Agrarian Technological Institute of Castilla and Leon (ITACyL), Ctra. Burgos Km 119, Finca Zamadueñas, 47071 Valladolid, Spain; jimpuliv@itacyl.es; 2 Endocrinology and Clinical Nutrition Research Center (IENVA), Faculty of Medicine, University of Valladolid, Av. Ramón y Cajal, 3, 47003 Valladolid, Spain; dluisro@saludcastillayleon.es (D.d.L.);

**Keywords:** bioaccessibility, digestion, dynamic, nutraceutic ingredient, gastrointestinal dynamic model

## Abstract

Efficient development of effective functional foods and nutraceuticals requires adequate estimation methods of the bioaccessibility of their bioactive compounds. Specially grain-based nutraceuticals and functional ingredients are often enriched in bound/low bioavailable bioactive phytochemicals. The objective of this work was to evaluate the differences in applying static or dynamic digestion models for the estimation of bioaccessibility of antioxidants present in cereal grain-based/fiber-rich ingredients produced using enzymatic hydrolysis and sprouting processes. Main liberated phenolic compounds, antioxidant activity (ABTS^•+^ and ORAC) and ferric reducing capacity were evaluated in the samples following three digestion protocols with differences based on their dynamism: static, semi-dynamic and dynamic. The samples digested with the dynamic method showed higher antioxidant and reducing capacities than those digested with the static and semi-dynamic protocols. The results obtained from the digests with the dynamic model showed a total phenol content (TPs) ranging from 1068.22 to 1456.65 μmol GAE 100 g^−1^ and antioxidant capacity values from 7944.62 to 15,641.90 μmol TE 100 g^−1^ (ORAC) and from 8454.08 to 11,002.64 μmol TE 100 g^−1^ (ABTS^•+^), with a reducing power ranging from 2103.32 to 2679.78 mmol Fe reduced 100 g^−1^ (FRAP). The dynamic character of the protocols used for developing bioactive cereal-based foods significantly affects the estimation of their bioaccessibility, probably giving a better approach to their potential bioavailability in in vivo systems.

## 1. Introduction

In the search for a healthier and more balanced diet, functional foods have gained importance and provided opportunities to improve health and prevent chronic diseases [1,2,3].

Cereal fiber is found mostly in the bran layer; fiber promotes digestive health by regulating bowel function and reduces the risk of chronic diseases [4,5,6,7]. On the other hand, most of the bioactive compounds present in those sources are in bound form and highly associated with dietary fiber, resulting in low bioaccessibility values. Nevertheless, these bioactive compounds have shown significant health effects, with phytosterols helping reduce serum LDL cholesterol levels and antioxidant polyphenols protecting against oxidative stress and inflammation [8,9,10,11].

In order to increase the health potential of cereal whole grains and bran, different biotechnological strategies have been attempted, based on germination and enzymatic hydrolysis, increasing the extractability of phenolic compounds [12,13,14]. Furthermore, the proposed biotechnological processes avoid high-temperature conditions occurring in other grain processes, conditions that have been reported to reduce polyphenol bioaccessibility [15].

Prior to being bioavailable, compounds are released from the food matrix and solubilized during digestion, becoming bioaccessible for further uptake and absorption [16]. Testing the bioaccessibility of bioactive compounds or nutrients is of vital importance when developing effective functional foods and nutraceuticals. However, in vivo studies for bioaccessibility and bioavailability determination are costly and constrained by ethical issues. In order to evaluate the bioaccessibility of nutraceuticals or functional foods, it is interesting to determine how they behave in the digestive system. However, this is a complex process until in vivo trials can be performed, and sometimes it is not an easy process [17]. For this reason, the development of a reliable intermediate method that simulates the human digestive system is interesting. Human digestive system replication in vitro allows for greater experimental controllability and reproducibility and also offers the possibility of performing preliminary studies without the need to perform in vivo methods.

In this regard, in vitro methods and models for simulated digestion have rapidly evolved in recent decades, in order to facilitate studies that provide evidence on the dynamics of the release of nutrients and bioactive compounds [18]. Static artificial digestion systems are the most widely used, due to their low cost and simplicity of implementation in any laboratory, without the need to purchase specific equipment [19]. Semi-dynamic and dynamic models provide a closer approach to physiological conditions, introducing new factors such as gradual addition of enzymes or gastric emptying gradients. This facilitates assessing the effects of food structure and better profiling the release of nutrients and bioactive compounds.

Despite logical limitations of in vitro models, these have facilitated screening studies of an important number of compounds and matrices in variable testing conditions. On the other hand, there are a large number of models published with diverse experimental conditions, which makes it difficult to compare the results from different studies. In this regard, an important effort to standardize static and semi-dynamic models was carried out by the International Network on Food Digestion (INFOGEST) project [20,21].

The protocol proposed by the COST action INFOGEST represents a significant advance in gastrointestinal digestion research, providing a standardized and reproducible method for the study of food digestion [22]. This method accurately simulates the physiological conditions of the human digestive tract, using a combination of digestive enzymes, gastrointestinal fluids and pH conditions to closely replicate the digestive processes. This approach allows a systematic and comparative assessment of how different foods, ingredients and formulations influence the digestibility, nutrient release and bioaccessibility of bioactive compounds [23]. Nevertheless, differences between static, semi-dynamic and dynamic models are unavoidable, and so are their practical applications.

It is important, therefore, to carry out studies comparing results from standardized methods using static, semi-dynamic and dynamic models, as proposed in this study, since agitation and movement through different phases can directly affect the bioaccesibility of compounds and their final bioactivity.

Therefore, the main objective of this study was to evaluate three digestion models, with differences in dynamism, based on the INFOGEST consensus protocols, in order to determine the differences on the results obtained and potential utility for bioaccessibility determination.

## 2. Materials and Methods

### 2.1. Chemicals

6-hydroxy-2,5,7,8-tetramethyl-2-carboxylic acid (Trolox), fluorescein, 2,2′-diazobis-(2-aminodinopropane)-dihydrochloride (AAPH), iron (III) chloride hexahydrate (FeCl_3_∙6H_2_O), Folin–Ciocalteu (FC) reagent, 2,20-azinobis 3-ethylbenzothiazoline-6-sulfonic acid (ABTS^•+^), 2,4,6-tripyridyl-triazine (TPTZ), ammonium molybdate, iron (II) sulfate heptahydrate (FeSO_4_∙7H_2_O), ascorbic acid, sodium hydroxide, sodium phosphate (NaH_2_PO_4_∙H_2_O), trichloroacetic acid, gallic acid (GA), ferulic acid, *p*-coumaric, acid hydroxybenzoic acid, potassium chloride, potassium phosphate monobasic, sodium bicarbonate, sodium chloride, magnesium chloride hexahydrate, ammonium carbonate, calcium chloride dihydrate, α-amylase, pepsin, pancreatin and bile salts were obtained from Sigma-Aldrich, Co. (St. Louis, MO, USA). Sulfuric acid, hydrochloric acid, sodium acetate and glacial acetic acid were provided by PanReac AppliChem (ITW Reagents, Darmstadt, Germany). Food-grade enzymes UltraFlo XL and Viscoferm were generously supplied by Novozymes (Bagsværd, Copenhagen, Denmark).

### 2.2. Materials

Raw materials, wheat grain (WG) and bran (WB) were generously supplied by Emilio Esteban, S.A. (Valladolid, Spain), and oat grain (OG) and hull (OH) were kindly provided by Sdad. Coop. Regional Ltda. Ribera del Duero (Burgos, Spain). Wheat (*Triticum aestivum* L.) was harvested in Valladolid in 2020, and bran was separated from endosperm by a dry milling process. Oat (*Avena sativa* L., *var. Chimene*) was harvested in Burgos in 2019–2020, and hulls were separated from the grain through a mechanical process under dry conditions. The raw materials were milled and stored in vacuum until analysis.

### 2.3. Sample Preparation

Wheat grain (WG) and oat grain (OG) were germinated using a previously described method [13]. The grains were sanitized by soaking them in water with 0.5% sodium hypochlorite (*v*/*v*) for 30 min at a ratio of 1:6 (*w*/*v*). After rinsing with water to neutralize the pH, the grains were placed in distilled water for 4 h. Sanitized grains were then transferred to trays and kept in a germination chamber (Snijders Scientific, Tilburg, The Netherlands) for 5 days at 21 °C with more than 90% of relative humidity. The resulting sprouts, SW and SO, were subjected to a high-pressure process (HPP) (Wave 6000/135, Hiperbaric, Burgos, Spain) at 600 MPa for 5 min and then freeze-dried (LyoBeta, Telstar, Barcelona, Spain).

Wheat bran (WB) and oat hulls (OH) were hydrolyzed by an enzymatic hydrolysis treatment, according to previously described method [13], with some modifications. WB and OH were resuspended in water at a 1:20 (*w*/*v*) ratio, and then the pH was corrected with malic acid to 5. Enzymes (UltraFlo XL for WB and Viscoferm for OH) were added at 1% of WB or OH dry weigh (*w*/*w*). The mixtures were incubated with agitation in a water bath at 47 °C over 20 h (Unitronic Vaivén C, Selecta S.A., Spain). After incubation, to inactivate the enzymes, the mixtures were heated at 95 °C for 5 min, followed by a high-pressure treatment (HPP) at 600 MPa for 5 min. Insoluble fraction of hydrolysates, EH-WB and EH-OH, was drained with a nylon filter (200 μm mesh) and freeze-dried (LyoBeta, Telstar, Barcelona, Spain).

All ingredients (SW, SO, EH-WB and EH-OH) were milled to a particle size of 0.5 mm. Subsequently, three different combinations of these ingredients were prepared in various proportions by weight, as described previously by authors [24]. Table 1 shows the different proportions by weight of the combined ingredients and Table S1 shows the proximal composition of the individual and combined ingredients.

### 2.4. Digestion

**Figure 1:**
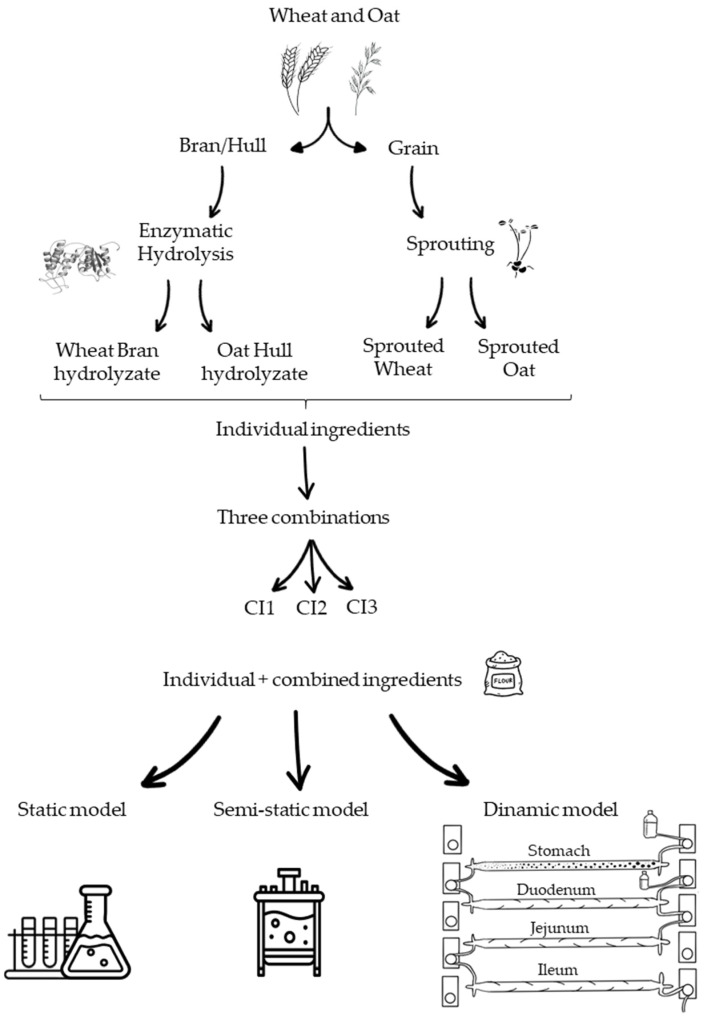
Scheme of digestion models of individual and combined ingredients.

The individual ingredients and the combined ingredients were digested by three different methods, static, semi-dynamic and dynamic, following and adapting the INFOGEST protocol [22] in each case (Figure 1). The simulated fluids during digestion were prepared according to the INFOGEST protocol (Table S2).

#### 2.4.1. Static Digestion Model

One gram of each ingredient was weighed into a 15 mL tube, and 1 mL of distilled water and 1 mL of simulated salivary fluid (SSF) were added. The tubes were incubated at 37 °C for 2 min. Then, 2 mL of simulated gastric fluid (SGF) was added, and pH was adjusted to 3 with 1 M HCl and incubated for 2 h at 37 °C. After this time, 4 mL of simulated intestinal fluid (SIF) was added, and pH was adjusted to 7 with 1 M NaHCO_3_ and incubated for 2 h at 37 °C. Finally, the enzymes were inactivated at 95 °C for 10 min.

#### 2.4.2. Semi-Dynamic Digestion Model

Sixty grams of ingredient was weighed, and 400 mL of distilled water and 60 mL of simulated salivary fluid were added. It was mixed and introduced into the equipment (Minifors 2, INFORS HT, Bottmingen, Switzerland) and incubated at 37 °C for 2 min. Subsequently, 500 mL of simulated gastric fluid was added, and the pH was adjusted to 3 with 1 M HCl and incubated for 2 h at 37 °C with shaking at 200 rpm. After this time, 1 L of simulated intestinal fluid was added, and pH was adjusted to 7 with 0.5 M NaHCO_3_ and incubated for 2 h at 37 °C with shaking at 300 rpm. Finally, various aliquots of the digest were taken, and the enzymes were inactivated at 95 °C for 10 min.

#### 2.4.3. Dynamic Digestion Model

The equipment was prepared with the solutions necessary for pH adjustment (HCl 1 M, NaHCO_3_ 0.5 M), and the different compartments, stomach, duodenum, jejunum and ileum, were filled with the corresponding simulated fluid (gastric and intestinal), leaving the stomach half full. Each compartment consisted of a plastic tube inside a cylinder, with hot water circulating through it to maintain the temperature during the process. The volume of each compartment was 100 mL for the stomach and 100 mL for the entire duodenum, jejunum and ileum. Six grams of sample were weighed, and 40 mL of distilled water and 6 mL of simulated salivary fluid were added, mixed well and incubated for 2 min at 37 °C. After that, the sample was introduced into the stomach and incubated at 37 °C for 2 min. Subsequently, the sample was introduced into the stomach compartment, and the next steps were started. The flow rate was 1 mL/min for the gastric phase and 2 mL/min for the intestinal phase. The pH adjustment was performed continuously using pH meters in each phase and adjustment pumps, maintaining pH 3 in the gastric phase and pH 7 in the intestinal phase, connected to an automatic controller. The sample was taken by collecting the output fluid at the end of the equipment from minute 120 to 330 min. After that, the enzymes were inactivated at 95 °C for 10 min.

Finally, all samples of the digests obtained in each model were freeze-dried (LyoBeta, Telstar, Barcelona, Spain) for subsequent analysis.

### 2.5. Extraction

One gram of digested sample was weighed and mixed with 10 mL of methanol:distilled water (50% *v*/*v*) pH 2. Subsequently, it was centrifuged (4000 rpm, 10 min, 4 °C), and the supernatant was filtered. Eight mL of methanol:distilled water (50% *v*/*v*) pH 2 was added over the precipitate, stirred and incubated again. Samples were centrifuged again, and the supernatant was filtered over the previous one. Finally, methanol:distilled water (50% *v*/*v*) pH 2 was added to a final volume of 20 mL and aliquoted. Samples were stored at −80 °C until future analysis.

### 2.6. Total Phenolics Content (TPs)

Total phenol content (TPs) was measured following the method reported by Slinkard and Singleton [25], utilizing the Folin–Ciocalteu phenol reagent. To quantify the TPs content in the samples, a standard curve was created using gallic acid (700–98 μM). The absorbance of the standards and samples was determined at 765 nm using a microplate reader (Fluostar Omega, BMG, Ortenberg, Germany). Results were expressed as μmol gallic acid equivalents (GAE) per 100 g dry matter (d.m.). All analyses were conducted in duplicate.

### 2.7. Determination of Phenolic Compounds by HPLC

Samples were filtered using a 0.22 μm nylon filter. An HPLC system (Agilent 1200, Agilent Technologies, Santa Clara, CA, USA) equipped with a DAD detector (Agilent G1315B) was employed to separate the phenolic compounds. Separation was performed on a Luna C18 column (250 mm × 2 mm i.d., 5 μm, Phenomenex, Torrance, CA, USA) at 25 °C. A 0.1% aqueous formic acid (solvent A) and 0.1% formic acid in acetonitrile (solvent B) were used as elution gradients. The flow rate applied was 0.4 mL/min, and the gradient program was as follows: 0 min, 8% B; 10 min, 23% B; 15 min, 50% B; 20 min, 50% B; 23 min, 100% B, with a subsequent re-equilibration step. The injection volume was 20 μL.

For quantification, authentic standard curves for gallic acid, hydroxybenzoic acid, *p*-coumaric acid and ferulic acid were prepared over a concentration range of 0.1 to 25 μg mL^−1^, exhibiting good linearity (R^2^ > 0.99). Results were expressed as mg per 100 g of sample (d.m.).

### 2.8. Total Antioxidant Capacity (TAC)

Total antioxidant capacity (TAC) was determined, for duplicate, with different methods: oxygen radical absorbance capacity (ORAC), radical cation scavenging activity ABTS^•+^ and ferric reducing antioxidant power (FRAP).

#### 2.8.1. Oxygen Radical Absorbance Capacity (ORAC)

The method described by Ou et al. [26], with some modifications, was followed to perform the ORAC assay. Phosphate buffer (75 mM, pH 7.4) was used to dilute the standard Trolox curve (7.5–210 μM) and samples. Twenty-five μL of the sample, Trolox standard and phosphate buffer (as blank) were mixed with 125 μL of fluorescein in a black 96-well microplate and incubated at 37 °C for 3 min. Then, the oxidation reaction was started after adding 25 μL of AAPH solution, and fluorescence was monitored for 150 min with an excitation filter at 485 nm and emission filter at 520 nm in a microplate reader (CLARIOstar Plus, BMG, Ortenberg, Germany). The areas under the fluorescein decay curves were plotted against Trolox concentration to calculate the results, which were expressed as μmol TE per 100 g of sample (d.m.).

#### 2.8.2. ABTS^•+^ Radical Cation Scavenging Activity

The ABTS^•+^ assay was performed according to the method described by Re et al. [27], with some modifications. Twenty microliters of the sample or standards was combined with 200 μL of ABTS^•+^ working solution in a 96-well microplate. After one hour of incubation, absorbance was measured at 734 nm using a microplate reader (Spectrostar Omega, BMG Ortenberg, Germany). A Trolox standard curve (210–7.5 μM) was used as standard. Results were expressed as μmol Trolox equivalents (TE) per 100 g of sample (d.m.).

#### 2.8.3. Ferric Reducing Antioxidant Power (FRAP)

FRAP assay was conducted following the method reported by Benzie and Strain [28], with minor modifications. Acetate buffer (300 mM, pH 3.6), TPTZ solution (10 mM in 40 mM HCl) and FeCl_3_∙6H_2_O solution (20 mM) in a 10:1:1 volume ratio were used to prepare the FRAP working solution. An FeSO_4_∙7H_2_O standard curve (400–3000 μM) was used for calibration. In Eppendorf tubes, twenty microliters of sample, standard or distilled water (as blank) were added. Then, 1.9 mL of the FRAP working solution was added to each tube, and they were stirred. After incubating the tubes for 5 min, absorbance was measured at 593 nm in a 96-well plate using a microplate reader (Spectrostar Omega, BMG Ortenberg, Germany). Results were expressed as mmol Fe equivalents (FeE) per 100 g of sample (d.m.).

### 2.9. Statistical Analysis

Data were reported as mean ± standard deviation and range. To determine differences between mean values, analysis of variance (ANOVA) and Duncan’s post hoc tests were conducted using the software Statgraphics Centurion XVIII^®^ (Statgraphics Technologies, Inc., The Plains, VA, USA). Significant differences were considered with a *p*-value of below 0.05.

## 3. Results

### 3.1. Digestion in Static Model

**Figure 2:**
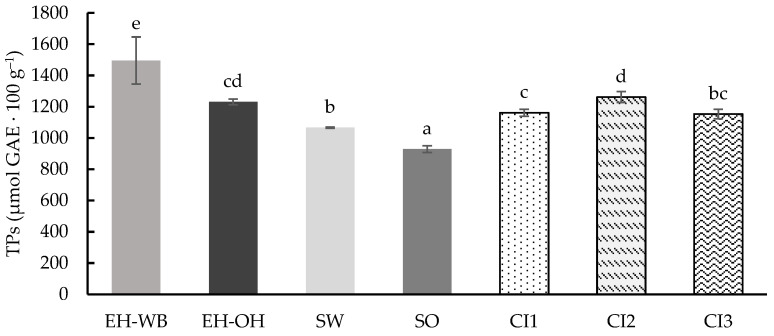
Total phenolic (TPs) content of the digests of the individual (EH-WB, EH-OH, SW and SO) and combined (CI1, CI2 and CI3) ingredients, following the static model. Results are expressed as μmol GAE 100 g^−1^ of d.m. Mean are values represented as bars, and standard deviations are represented as error bars. Different letters indicate significant differences from each other (one-way ANOVA, post hoc Duncan’s test, *p* < 0.05). Abbreviations: wheat bran hydrolysate (EH-WB), oat hull hydrolysate (EH-OH), sprouted wheat (SW), sprouted oat (SO), combined ingredient 1, 2 and 3 (CI1, CI2, CI3).

Total phenolic compound (TPs) content of the samples digested following the static model are shown in Figure 2. The values obtained ranged from 928.93 to 1495.76 μmol GAE 100 g^−1^, showing significant differences (*p* < 0.05) between them. Digested samples of the ingredients obtained by enzymatic hydrolysis (EH-WB and EH-OH) showed higher contents than those obtained by germination (SW and SO). According to the type of matrix, ingredients from wheat showed higher TPs than those from oats. Digested combined ingredients (CI1, CI2 and CI3) reflected the proportion of TPs as present in each individual ingredient used for their formulation, with the combined ingredient CI2, with a higher proportion of enzymatic hydrolysates (EH-WB and EH-OH), presenting higher TP values than CI1 and CI3.

HPLC was used to characterize and determine the content of the main phenolic compounds in the digests of the different individual ingredients (EH-WB, EH-OH, SW and SO) and the combined ingredients (CI1, CI2 and CI3). Figure S1 shows the chromatograms of the identified compounds. Table 2 shows the results obtained for the phenolic compounds characterized and determined in the different digested samples, which ranged from 0.15 to 1.08 mg 100 g^−1^ for coumaric acid and from 0.16 to 3.26 mg 100 g^−1^ for ferulic acid, showing significant differences (*p* < 0.05). Similarly to TPs, digests from wheat samples showed higher content of these phenolic compounds; however, the enzymatic hydrolysis treatment did not favor their concentration, while germination did produce a higher amount of these compounds. The digests of the different combinations of ingredients, unlike the content in TPs, do not follow the proportionality in which the individual ingredients are found, with the digest of the CI2 ingredient standing out as containing the highest amount of both characterized phenolic compounds. This could be related to a possible interaction between phenolic compounds and other macromolecules, such as proteins or fiber.

The total antioxidant capacity (TAC) was determined following assays to evaluate its activity against different radicals (ORAC and ABTS^•+^), and the reducing power was determined with the FRAP assay.

**Figure 3:**
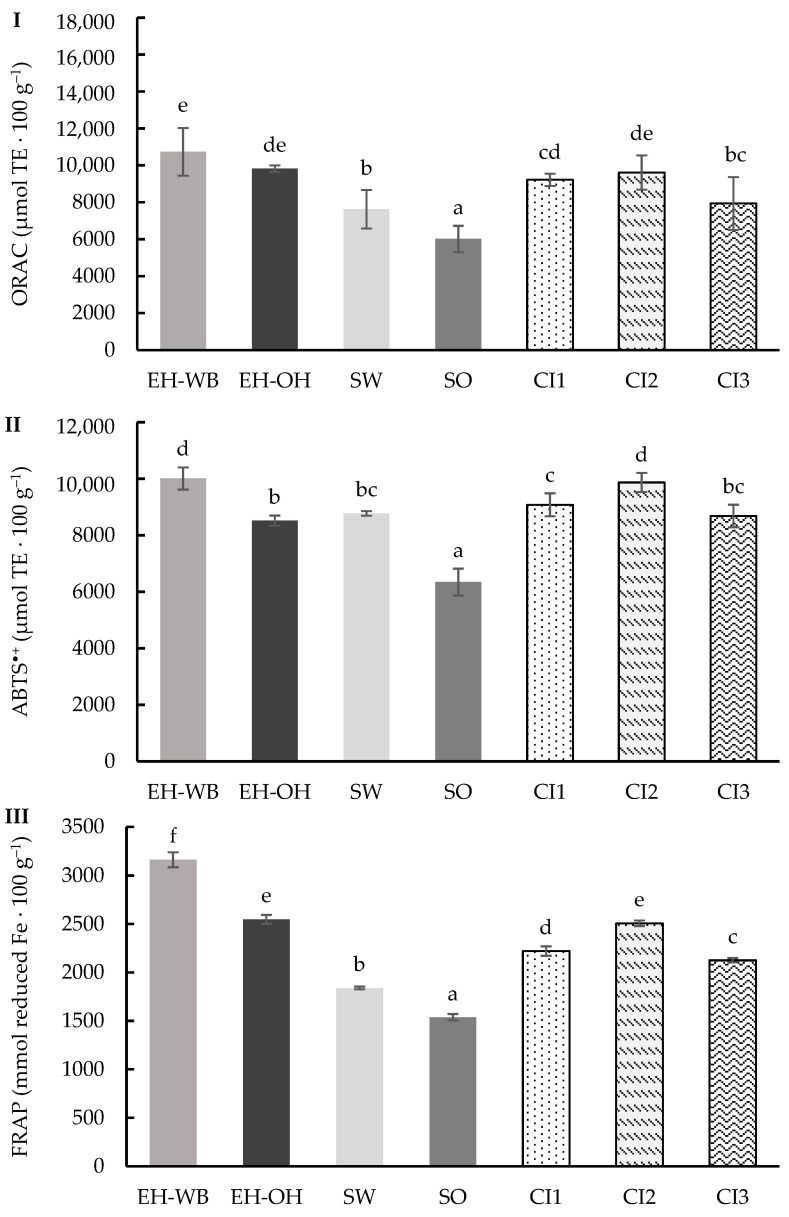
ORAC (**I**), ABTS^+^ (**II**) and FRAP (**III**) values of the digests of the individual (EH-WB, EH-OH, SW and SO) and combined (CI1, CI2 and CI3) ingredients following the static model. Results are expressed as μmol TE 100 g^−1^ of sample and mmol reduced Fe 100 g^−1^. Mean values are represented as bars, and standard deviations are represented as error bars. Different letters indicate significant differences from each other (one-way ANOVA, post hoc Duncan’s test, *p* < 0.05). Abbreviations: wheat bran hydrolysate (EH-WB), oat hull hydrolysate (EH-OH), sprouted wheat (SW), sprouted oat (SO), combined ingredient 1, 2 and 3 (CI1, CI2, CI3).

The ORAC method enabled the evaluation, in the digests of the individual and combined ingredients, of the capacity to neutralize peroxyl radicals generated by AAPH through the transfer of hydrogen atoms (Figure 3I) [29]. The results obtained ranged from 6017.37 to 10,730.39 μmol TE 100 g^−1^, showing significant differences (*p* < 0.05). The digests from the samples from the enzymatic hydrolysis treatment gave higher activity against the peroxyl radicals than the digests from the germinated samples. The digests from the wheat ingredient samples showed higher activity against the peroxyl radicals than the oat ingredients. The digests from the combination of ingredients maintained the proportionality of the values obtained in the digests of the individual ingredients.

Also, ABTS^•+^ radical activity of the digests of the different ingredients was determined (Figure 3II). This assay evaluates the ability of antioxidants to participate in electron transfer reactions [30]. Significant differences (*p* < 0.05) were observed between the different ingredients, with values ranging from 6345.17 to 10,010.76 μmol TE 100 g^−1^. The results obtained for the digests of the hydrolyzed ingredients were higher than those obtained for the digests of the germinated ingredients. According to the type of raw material, the digests of wheat ingredients showed higher activity against the ABTS^•+^ radical than the digests of oat ingredients. The digests of the combined ingredients showed values between 8684.06 and 9871.09 μmol TE 100 g^−1^, showing significant differences (*p* < 0.05) between them and maintaining the proportion in which each individual ingredient was found, with the CI2 digest especially interesting.

The reducing power of the different digests was determined using the FRAP assay, and the results obtained are shown in Figure 3III. This assay measures the capacity to reduce iron (III) ions, which indicates the potential of the samples to neutralize free radicals. The values obtained ranged from 1538.10 to 3160.94 mmol reduced Fe 100 g^−1^, showing significant differences (*p* < 0.05) between samples. Similar to the previous markers, the digests from the hydrolyzed ingredients showed higher reducing power than the sprouts, and it was also higher in the digests from the wheat samples than from the oats. Likewise, the digests from the combined ingredients showed a reducing power according to the proportion in which the individual ingredients were found.

### 3.2. Digestion in Semi-Dynamic Model

**Figure 4:**
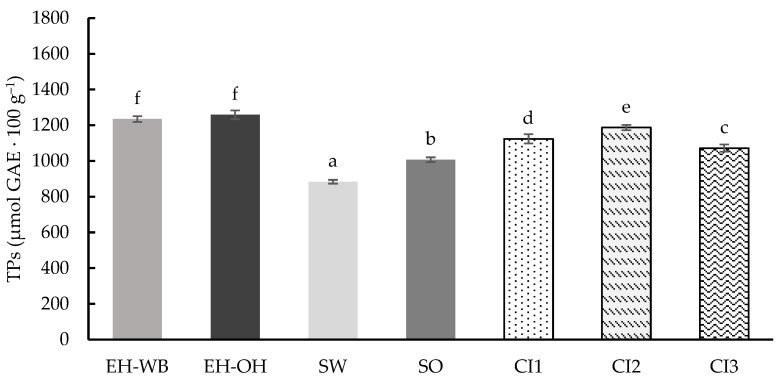
Total phenolics (TPs) content of the digests of the individual (EH-WB, EH-OH, SW and SO) and combined (CI1, CI2 and CI3) ingredients following the semi-dynamic model. Results are expressed as μmol GAE 100 g^−1^ of d.m. Mean values are represented as bars, and standard deviations are represented as error bars. Different letters indicate significant differences from each other (one-way ANOVA, post hoc Duncan’s test, *p* < 0.05). Abbreviations: wheat bran hydrolysate (EH-WB), oat hull hydrolysate (EH-OH), sprouted wheat (SW), sprouted oat (SO), combined ingredient 1, 2 and 3 (CI1, CI2, CI3).

As with samples subjected to digestion with the static model, the total phenolic compound (TPs) content was determined in the samples after digestion following the semi-dynamic model (Figure 4). In this case, the values obtained ranged from 884.21 to 1258.66 μmol GAE 100 g^−1^, showing significant differences (*p* < 0.05) between them. Similarly, to the static model, the digested samples of the hydrolyzed ingredients showed higher TPs content than those digested from the germinated ingredients. However, if we compare the matrices of the ingredients, the content of TPs is higher in those from oats compared to those from wheat. On the other hand, the digests of the combined ingredients showed a TPs content according to the proportion of the individual ingredients in each combination, with CI2 being the combined ingredient with the highest TPs content.

Table 3 shows the values obtained for the phenolic compounds characterized in the different digests by HPLC. Figure S1 shows the chromatograms of the identified compounds. The values for coumaric acid ranged from 0.02 to 1.24 mg 100 g^−1^, and for ferulic acid, from 0.07 to 0.87 mg 100 g^−1^, showing significant differences (*p* < 0.05). Digested samples from wheat showed higher values than digested samples from oats. The germination process allows obtaining better values for the characterized compounds than after the hydrolysis process. The results observed for the digests of the combined ingredients were low, being under the limit of detection in some cases. As it occurred with the static digestion model, it is possible that an interaction between phenolic compounds and other macromolecules, such as proteins or fiber, could exist due to the processes of germination and hydrolysis.

**Figure 5:**
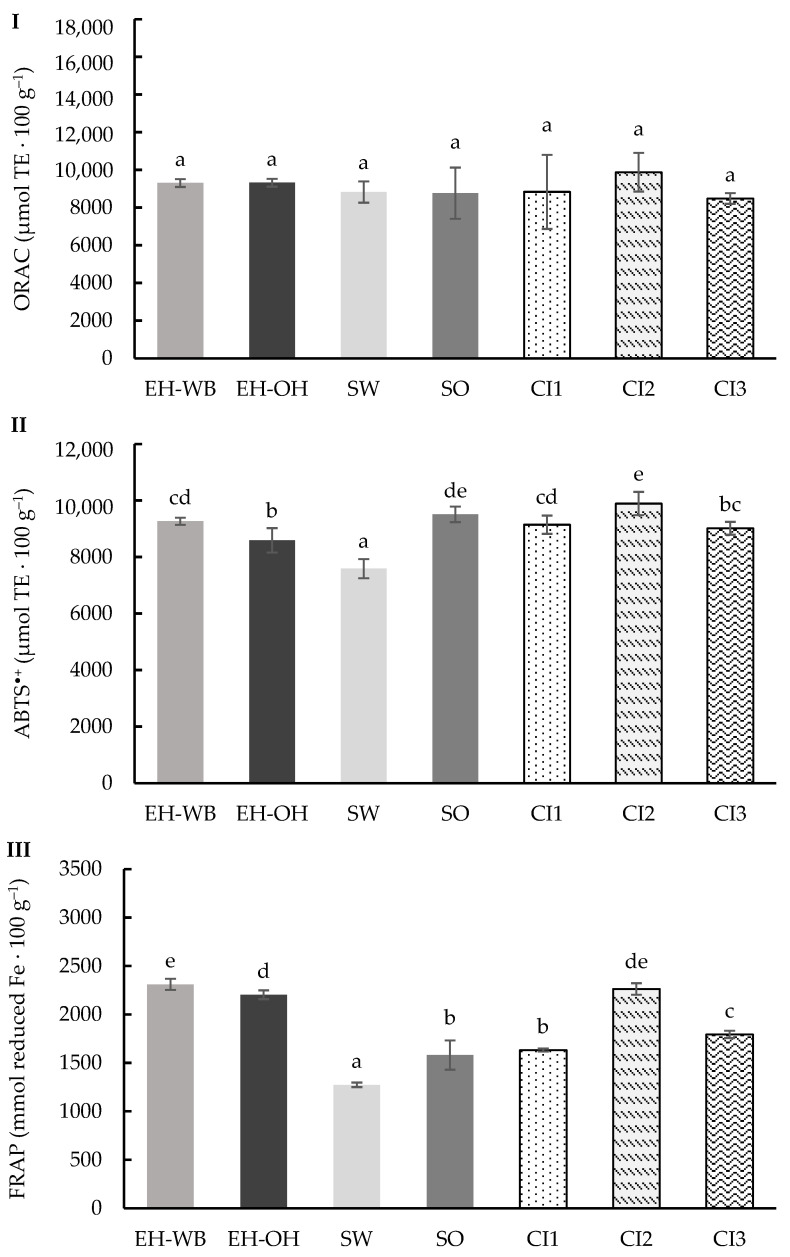
ORAC (**I**), ABTS^•+^ (**II**) and FRAP (**III**) values of the digests of the individual (EH-WB, EH-OH, SW and SO) and combined (CI1, CI2 and CI3) ingredients following the semi-dynamic model. Results are expressed as μmol TE 100 g^−1^ of sample and mmol reduced Fe 100 g^−1^. Mean values are represented as bars, and standard deviations are represented as error bars. Different letters indicate significant differences from each other (one-way ANOVA, post hoc Duncan’s test, *p* < 0.05). Abbreviations: wheat bran hydrolysate (EH-WB), oat hull hydrolysate (EH-OH), sprouted wheat (SW), sprouted oat (SO), combined ingredient 1, 2 and 3 (CI1, CI2, CI3).

The total antioxidant capacity (TAC) in the digests was also determined following the semi-dynamic model using ORAC, ABTS^•+^ and FRAP assays (Figure 5).

The results obtained after evaluating the digests of the individual and combined ingredients with the ORAC method are shown in Figure 5I. The values obtained ranged from 8483.12 to 9878.20 μmol TE 100 g^−1^, with no significant differences (*p* < 0.05) between them. The results obtained showed that the digests of the hydrolyzed ingredients were found to have a higher activity against the AAPH radical than the digests of the germinated ingredients, with no differences observed between the ingredient matrices (wheat or oat). Digests of the combined ingredients showed values ranging from 8483.12 to 9878.20 μmol TE 100 g^−1^, with CI2 showing the highest activity against the radical.

Results obtained for the activity against the ABTS^•+^ radical are shown in Figure 5II. Significant differences (*p* < 0.05) are observed between the values obtained, which ranged from 7590.66 to 9894.70 μmol TE 100 g^−1^. In this case, the matrix or the treatment used to obtain the ingredients did not show a clear pattern. However, the digests of the combined ingredients did maintain the proportionality of the individual ingredients, with the digest of CI2 being the most remarkable.

Finally, the reducing power of the different digests of the individual and combined ingredients was determined (Figure 5III). Significant differences (*p* < 0.05) were observed between the values obtained, which ranged from 1274.20 to 2310.84 mmol reduced Fe 100 g^−1^. The digests of the hydrolyzed ingredients showed higher reducing power compared to the sprouted ingredients, while the source matrix did not show any tendency. The values obtained in the digests of the combined ingredients maintained the ratio in which the individual ingredients are found, especially CI2.

### 3.3. Digestion in Dynamic Model

**Figure 6:**
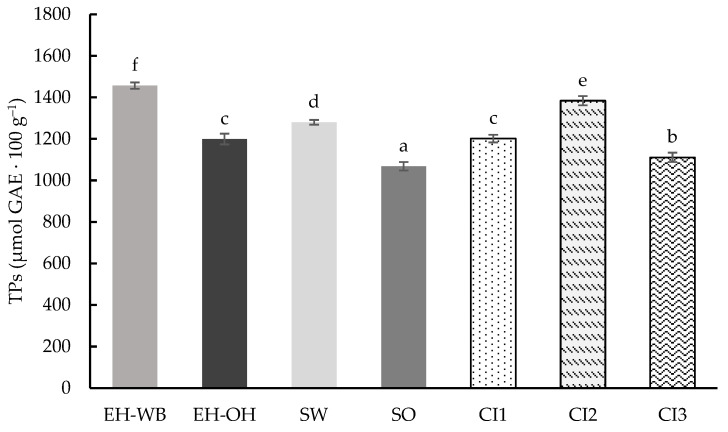
Total phenolics (TPs) content of the digests of the individual (EH-WB, EH-OH, SW and SO) and combined (CI1, CI2 and CI3) ingredients following the dynamic model. Results are expressed as μmol GAE 100 g^−1^ of d.m. Mean values are represented as bars, and standard deviations are represented as error bars. Different letters indicate significant differences from each other (one-way ANOVA, post hoc Duncan’s test, *p* < 0.05). Abbreviations: wheat bran hydrolysate (EH-WB), oat hull hydrolysate (EH-OH), sprouted wheat (SW), sprouted oat (SO), combined ingredient 1, 2 and 3 (CI1, CI2, CI3).

Similar to the previous digestion models, the content of phenolic compounds was determined in the digestate samples following the dynamic model (Figure 6). Significant differences (*p* < 0.05) were observed between the values obtained, which ranged from 1068.22 to 1456.65 μmol GAE 100 g^−1^. Digests of ingredients from wheat showed higher amounts of TPs than those from oats. The hydrolysis treatment favored the release of phenolic compounds to a greater extent than the germination treatment. The digests of the combined ingredients maintained the proportion of the individual ingredients in each combination, with CI2 having a higher content of TPs.

The results obtained for the phenolic compounds characterized by HPLC in the different digests are shown in Table 4. Figure S1 shows the chromatograms of the identified compounds. Coumaric acid ranged from 0.01 to 3.36 mg 100 g^−1^, and ferulic acid from 1.44 to 28.57 mg 100 g^−1^, showing significant differences (*p* < 0.05) between the values obtained. As it occurred with the TPs results, the digests of the ingredients obtained from wheat showed a higher content of the characterized compounds than the ingredients obtained from oat. The digests of the combined ingredients do not reflect the proportions of the individual ingredients. Notably, the digest of the CI2 ingredient has the highest concentration of identified phenolic compounds.

**Figure 7:**
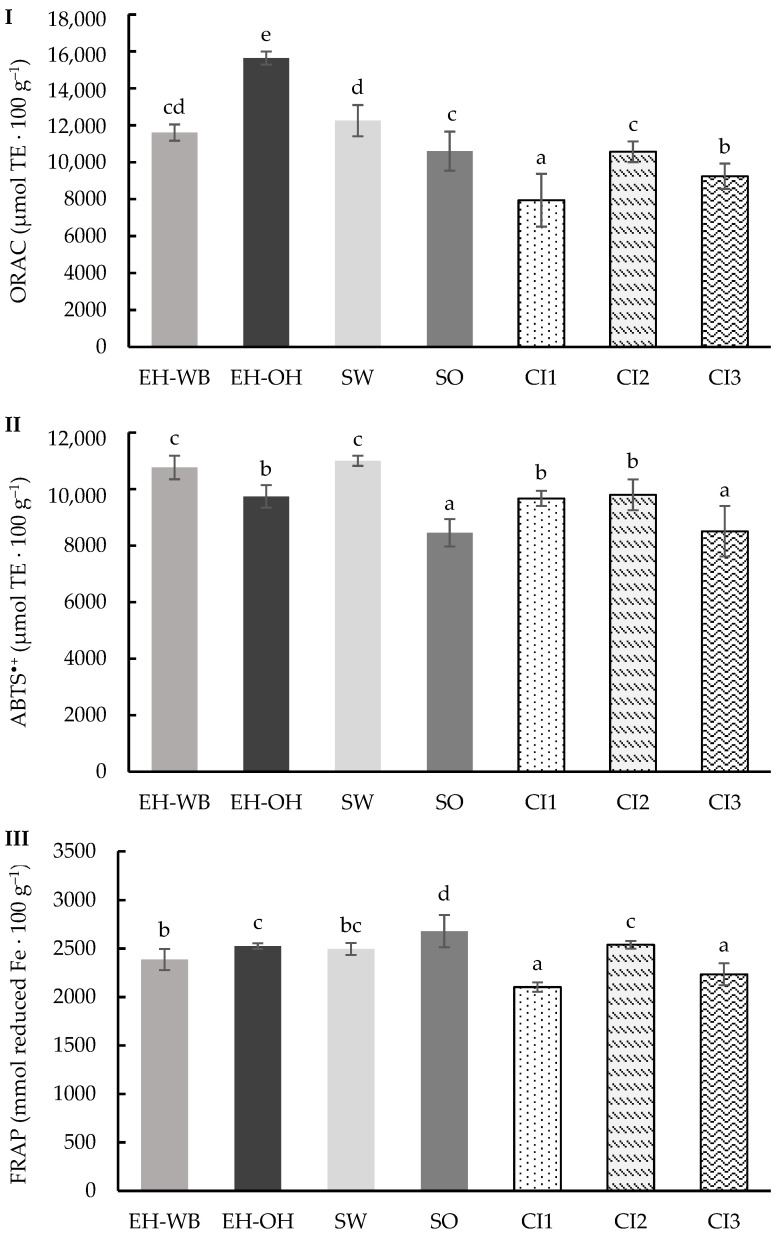
ORAC (**I**), ABTS^•+^ (**II**) and FRAP (**III**) values of the digests of the individual (EH-WB, EH-OH, SW and SO) and combined (CI1, CI2 and CI3) ingredients following the dynamic model. Results are expressed as μmol TE 100 g^−1^ of sample and μmol reduced Fe 100 g^−1^. Mean values are represented as bars, and standard deviations are represented as error bars. Different letters indicate significant differences from each other (one-way ANOVA, post hoc Duncan’s test, *p* < 0.05). Abbreviations: wheat bran hydrolysate (EH-WB), oat hull hydrolysate (EH-OH), sprouted wheat (SW), sprouted oat (SO), combined ingredient 1, 2 and 3 (CI1, CI2, CI3).

Furthermore, ORAC, ABTS^•+^ and FRAP assays were used to determine the total antioxidant capacity (TAC) of the digests following the dynamic model. Figure 7I shows the results obtained for the activity against the peroxyl radicals of the different digests, determined by ORAC assay. Significant differences (*p* < 0.05) are observed between the values obtained, which ranged from 7944.62 to 15,641.90 μmol TE 100 g^−1^. The type of raw material of the ingredients or the treatment carried out to obtain them does not show a clear trend in the results obtained. In relation to the digests of the combined ingredients, the results ranged from 7944.62 to 10,569.33 μmol TE 100 g^−1^, especially CI2.

Figure 7II shows the results obtained in the ABTS^•+^ radical activity test. The values obtained ranged from 8454.08 to 11,002.64 μmol TE 100 g^−1^, showing significant differences (*p* < 0.05) between the results. The type of raw material showed differences between the results, with the highest ABTS^•+^ radical activity in the digested samples of the wheat-based ingredients. However, the type of treatment applied to the ingredients showed no differences. In the same way as the previous markers, the results obtained in the digests of the combined ingredients maintain the proportion of the individual ingredients, in particular CI1 and CI2, with no differences between them.

Finally, the reducing power of the different digests of the individual and combined ingredients was evaluated (Figure 7III). The values obtained ranged from 2103.32 to 2679.78 mmol reduced Fe 100 g^−1^, showing significant differences (*p* < 0.05) between the results. The digests of the ingredients from oat showed higher reducing power than those from wheat. The treatment applied to the ingredients with the highest reducing power was the germination treatment. The digests of the combined ingredients obtained values from 2103.32 to 2539.39 mmol reduced Fe 100 g^−1^, especially the combined ingredient CI2.

The different digestion models were compared in each assay, in order to observe possible differences between them (Figure S2). The static and semi-dynamic models showed no differences between them; however, the dynamic model obtained the highest values, with significant differences (*p* < 0.05) in all assays. This suggests that the dynamic digestion model could be a potential model to determine the bioaccessibility of bioactive compounds in an approximation of the human digestion process.

## 4. Discussion

Different biotechnological strategies have been developed in the research for new ingredients with functional properties. Some of these options are based on the hydrolysis of the bran or hulls of cereals or the germination of whole grains [13,31]. However, the combination of different matrices has been found to be a promising strategy [24]. Furthermore, during the development of these ingredients, it is important to assess their bioaccessibility. For this, it is necessary to develop an in vitro process that allows an approach to the human digestive system, because in vivo methods are complex and difficult to perform [17], the INFOGEST protocol being a good starting point [22]. For this reason, three in vitro digestion models were evaluated to observe the differences related to the determination of bioaccessibility.

On the one hand, regarding the content of total phenols (TPs), the results obtained after digestion of the ingredients produced by hydrolysis (EH-WB and EH-OH) showed similar values to those previously reported [13,31]. Hydrolysis with enzymes enhances the release of phenolic compounds linked to other structures such as cellulose, lignin and proteins, which are the constituents of the bran structure [32]. The germination process produces physiological and structural changes in the grains, due to the degradation of the structures by the action of endogenous enzymes. This promotes the release of phenolic compounds, leading to an increase in the content of TPs [33,34].

The values obtained in TPs after digestion of the sprouted ingredients (SW and SO) showed higher values than previously reported by our group [13,24] and other authors, too [35,36]. This could be related to the varietal effect of the grains, to the conditions used during germination, as well as to the action of the enzymes involved in digestion, which may favor the release of phenolic compounds and their absorption throughout the digestive system [37]. The results obtained after digestion of the combined ingredients (CI1, CI2 and CI3) were similar to those previously reported by the authors [24], maintaining the proportion of the individual ingredients.

When comparing the content of TPs obtained in the different digestion models, significant differences are observed between them (Figure S2), especially in the dynamic model, showing a high content of TPs. This could be due to a higher contact of the food with the different phases of the process, and that, using a large volume, the release of phenolic compounds associated with the macromolecules that constitute the food matrix is favored.

In the characterization of phenolic compounds by HPLC, it has been observed that results are lower than those previously reported by the authors [13,31]. This reduction of phenolic compounds was probably due to the degradation of phenolic compounds and the production of secondary metabolites during the digestion process [38]. Comparing the three digestion models, similar to the TPs content, significant differences (*p* < 0.05) are observed between them, highlighting the dynamic model (Figure S2), in which, being more in contact with the phases of the process due to the large volume used, the phenolic compounds are released from the different matrices comprising the food.

On the other hand, ORAC, ABTS^•+^ and FRAP assays were used to determine the antioxidant capacity of the samples digested with the different digestion models.

The values obtained for the digests of the hydrolyzed ingredients (EH-WB and EH-OH) obtained in the assay against the AAPH radical (ORAC) were similar to results previously reported by the group [24,31]. The results of the digestion of the hydrolyzed by-products against the ABTS^•+^ radical were higher than the results previously obtained [24,31]. This increase can be associated with the degradation of the macromolecules of the food matrix and the production of other metabolites, such as peptides, which also present an affinity with the ABTS^•+^ radical due to their capacity to donate hydrogen atoms to free radicals [39]. The values obtained for the reducing power (FRAP) of the EH-WB and EH-OH digests were lower than those previously reported [24].

The results obtained in the ORAC assay for the digests of the germinated grains (SW and SO) were higher than those obtained previously by the group [13,24], due to the digestion process promoting the release of compounds that are linked to other macromolecules, and also by other authors [35], due to the varietal effect and the germination process. Results obtained from SW and SO digests against the ABTS^•+^ radical, similar to the digests of hydrolyzed ingredients, were higher than the results previously reported [13,24], which could be associated with the production of metabolites with antioxidant capacity, such as peptides. The reducing power (FRAP) observed in the digests of the germinated grains was higher than those previously obtained by the group [13,24].

Finally, regarding the digests of the combined ingredients (CI1, CI2 and CI3), the results obtained in the ORAC assay are slightly higher than those observed previously in digestion [24]. This increase, as we have seen previously, is associated with the degradation of the structure of the macromolecules and the release of compounds associated with them. The results obtained in the static and semi-dynamic models maintained the proportion of the individual ingredients, while the dynamic model showed a reduction of 20–40% with respect to the expected value.

The values obtained for the digests of the combined ingredients against the ABTS^•+^ radical is double the previously observed without digestion [24]. These results are similar to those observed in the ORAC assay. The degradation of macromolecules that occurs during the digestion process produces smaller metabolites. Some of these metabolites, such as peptides, have a high antioxidant capacity and are able to react with the ABTS radical. The results observed for the reducing power (FRAP) of the digested combined ingredients are lower than previously observed [24]. In the ABTS^•+^ and FRAP assays, no substantial differences are observed between the expected and real values of the different ingredient combinations, maintaining the proportion of each individual ingredient.

Comparing the results obtained for the antioxidant capacity in these models (Figure S2), it was observed that the digestion with the dynamic model showed the highest antioxidant capacity compared to the other two digestion models. The values observed in these assays were higher than those previously reported by other authors [38,40,41,42]. The differences found are mainly related to the methodology used during the digestion process, as well as to the varietal differences of the raw materials.

The results have shown than there are important variations in the digestion-mimicking conditions, such as the number of digestion steps, type, amount and release of digestive enzymes, the composition of fluids/electrolyte solutions, duration and pH of each digestion phase, and agitation, which directly affect the results obtained. These variations may prevent comparison of the results among different studies. In our case, digestion with the dynamic model resulted in higher bioaccessiblity of the bioactive (antioxidant) compounds.

Dynamic systems allowed the recovery of a higher concentration of phenolics, as compared to the static and semi-dynamic models (Figure S2), and these differences are reflected in the concentration of certain compounds, such as Ferulic and Cumaric acids, and indirectly in the antioxidant capacity of the samples. For this reason, in order to understand and predict human physiological digestion, it is important to consider dynamic systems to be closed to real conditions.

Our study has some limitations. Despite the dynamic models’ improvements, they still fall short of capturing the complex interplay of factors such as gut microbiota, hormonal influences and individual variability. Furthermore, in vitro digestion methods, while cost-effective and less ethically constrained, cannot completely replace in vivo studies. The absence of key physiological parameters limits the full understanding of nutrient absorption and metabolism. 

However, our design has some strengths. One major strength is its standardization of in vitro digestion models, particularly in the context of nutrient and bioactive compound bioaccessibility from food matrices. This ensures reproducibility and comparability of results across different laboratories, facilitating the understanding of food digestion and the bioavailability of nutrients.

Additionally, the use of a dynamic digestion model offers a more physiologically relevant simulation of the human gastrointestinal process compared to static models. This enhances the accuracy of bioaccessibility estimates, making the study outcomes more translatable to real-world settings.

## 5. Conclusions

The results obtained indicate that the INFOGEST protocol shows differences associated with the in vitro digestion models. As far as we know, this is the first study to compare three in vitro digestion models using the same protocol.

The digestion process using a dynamic model promotes more contact of the food with the different phases of digestion (oral, gastric and intestinal). This results in the degradation of structures and the fragmentation of macromolecules into smaller molecules, thus releasing compounds that were linked to them. These compounds, such as phenolic compounds, have biological activity and, according to various studies, have antioxidant, anticarcinogenic, anti-inflammatory, antidiabetic and cardioprotective properties.

This study shows that the development of a dynamic digestion model could be a useful strategy to evaluate the bioaccessibility of foods helping to tailor formulations for in vivo studies.

## Figures and Tables

**Table 1:** Formula of combined nutraceutical ingredients.

	Combined Ingredient 1 (CI1)	Combined Ingredient 2 (CI2)	Combined Ingredient 3 (CI3)
**EH-WB**	1	2	1
**EH-OH**	1	2	1
**SW**	1	1	2
**SO**	1	1	2

Abbreviations: wheat bran hydrolysate (EH-WB), oat hull hydrolysate (EH-OH), sprouted wheat (SW) and sprouted oat (SO).

**Table 2:** Phenolic compounds quantified by HPLC in digest samples, following the static model. Results are expressed as mg 100 g^−1^ of sample. Different letters indicate significant differences (*p* < 0.05).

	Cumaric Acid	Ferulic Acid
EH-WB	0.33 ± 0.07 ^b^	0.16 ± 0.06 ^a^
EH-OH	0.15 ± 0.00 ^a^	<LOD
SW	1.08 ± 0.01 ^e^	3.26 ± 0.05 ^e^
SO	<LOD	<LOD
CI1	0.63 ± 0.07 ^c^	1.62 ± 0.05 ^c^
CI2	0.86 ± 0.09 ^d^	1.93 ± 0.17 ^d^
CI3	0.58 ± 0.01 ^c^	0.60 ± 0.05 ^b^

Abbreviations: wheat bran hydrolysate (EH-WB), oat hull hydrolysate (EH-OH), sprouted wheat (SW), sprouted oat (SO), combined ingredient 1, 2 and 3 (CI1, CI2, CI3), limit of detection (LOD).

**Table 3:** Phenolic compounds quantified by HPLC in digest samples following the semi-dynamic model. Results are expressed as mg 100 g^−1^ of sample. Different letters indicate significant differences (*p* < 0.05).

	Cumaric Acid	Ferulic Acid
EH-WB	0.02 ± 0.01 ^a^	0.07 ± 0.03 ^a^
EH-OH	<LOD	<LOD
SW	1.24 ± 0.16 ^d^	0.87 ± 0.02 ^d^
SO	0.11 ± 0.06 ^b^	<LOD
CI1	0.73 ± 0.05 ^c^	0.20 ± 0.04 ^b^
CI2	<LOD	0.27 ± 0.02 ^c^
CI3	<LOD	<LOD

Abbreviations: wheat bran hydrolysate (EH-WB), oat hull hydrolysate (EH-OH), sprouted wheat (SW), sprouted oat (SO), combined ingredient 1, 2 and 3 (CI1, CI2, CI3), limit of detection (LOD).

**Table 4:** Phenolic compounds quantified by HPLC in digest samples following the dynamic model. Results are expressed as mg 100 g^−1^ of sample. Different letters indicate significant differences (*p* < 0.05).

	Cumaric Acid	Ferulic Acid
EH-WB	3.36 ± 0.02 ^e^	21.84 ± 0.00 ^e^
EH-OH	0.80 ± 0.47 ^c^	3.74 ± 0.46 ^b^
SW	0.01 ± 0.00 ^a^	28.57 ± 0.04 ^f^
SO	0.24 ± 0.00 ^b^	1.44 ± 0.00 ^a^
CI1	0.77 ± 0.04 ^c^	18.39 ± 0.17 ^d^
CI2	1.39 ± 0.02 ^d^	22.10 ± 0.35 ^e^
CI3	0.26 ± 0.07 ^b^	11.97 ± 0.20 ^c^

Abbreviations: wheat bran hydrolysate (EH-WB), oat hull hydrolysate (EH-OH), sprouted wheat (SW), sprouted oat (SO), combined ingredient 1, 2 and 3 (CI1, CI2, CI3), limit of detection (LOD).

## Data Availability

The original contributions presented in the study are included in the article, further inquiries can be directed to the corresponding author.

## Supplementary Materials

The following supporting information can be downloaded at: https://www.mdpi.com/article/10.3390/antiox13101244/s1, Table S1: Proximal composition of individual (EH-WB, EH-OH, SW and SO) and combined (CI1, CI2 and CI3) ingredients. Values were expressed as g 100 g^−1^ of dry matter; Table S2: Composition of the simulated fluids employed in the digestions; Figure S1: Chromatograms for the identified phenolic compounds in the digests of the individual (EH-WB, EH-OH, SW and SO) and combined (CI1, CI2 and CI3) ingredients following the static (**I**), semi-dynamic (**II**) and dynamic (**III**) model: 1 = cumaric acid, 2 = ferulic acid. Abbreviations: wheat bran hydrolysate (EH-WB), oat hull hydrolysate (EH-OH), sprouted wheat (SW), sprouted oat (SO), combined ingredient 1, 2 and 3 (CI1, CI2, CI3); Figure S2: Comparison of the different models of digestion according to the assays of Total Phenolics (TPs, **I**), cumaric acid (**II**), ferulic acid (**III**), ORAC (**IV**), ABTS (**V**) and FRAP (**VI**). Mean values represented as bars, and standard deviations represented as error bars. Different letters indicate significant differences from each other (one-way ANOVA, post hoc Duncan’s test, *p* < 0.05).
