# Supplementary material for: Comparative Bioaccesibility Study of Cereal-Based Nutraceutical Ingredients Using INFOGEST Static, Semi-Dynamic and Dynamic In Vitro Gastrointestinal Digestion
Source: Antioxidants (Basel). 2024 Oct 16;13(10):1244. doi: 10.3390/antiox13101244 (PMC11505457; doi:10.3390/antiox13101244)
Supplement: Supplementary file 1 [file antioxidants-13-01244-s001.zip › antioxidants-3246958-supplementary.pdf]

Figure S1. Chromatograms for the identified phenolic compounds in the digests of the individual (EH-WB, EH-OH, SW and SO) and combined (CI1, CI2 and CI3) ingredients following the static (**I**), semi-dynamic (**II**) and dynamic (**III**) model: 1 = cumaric acid, 2 = ferulic acid. Abbreviations: wheat bran hydrolysate (EH-WB), oat hull hydrolysate (EH-OH), sprouted wheat (SW), sprouted oat (SO), combined ingredient 1, 2 and 3 (CI1, CI2, CI3).

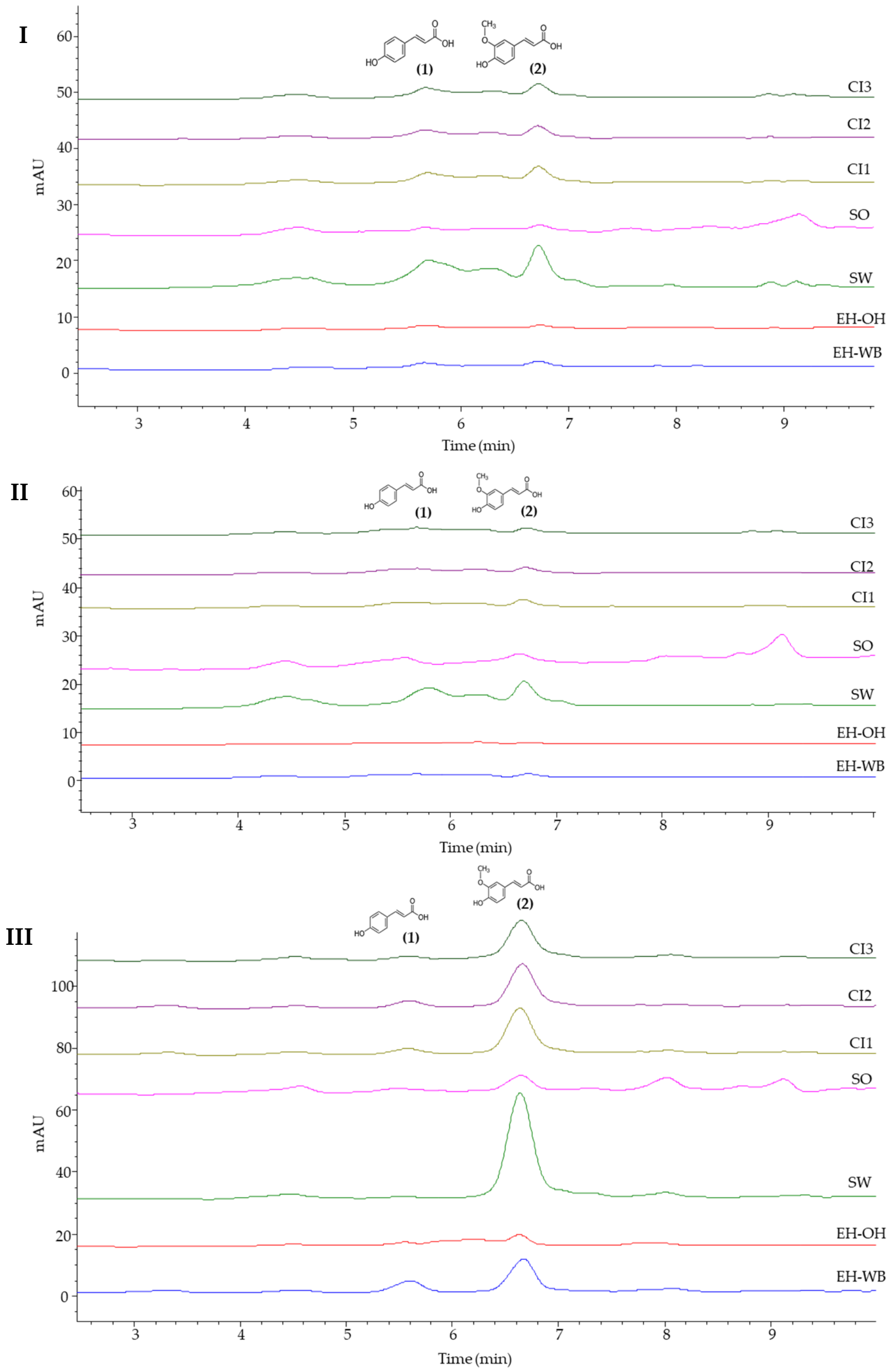

Table S1. Composition of the simulated fluids employed in the digestions.

	SSF	SGF	SIF
**KCl**	15.1 mM	6.9 mM	6.8 mM
**KH_2PO_4**	3.7 mM	0.9 mM	0.8 mM
**$NaHCO_3$**	13.6 mM	25 mM	85 mM
**NaCl**	-	47.2 mM	38.4 mM
**$MgCl_2(H_2O)_6$**	0.15 mM	0.12 mM	0.33 mM
**$(NH_4)_2CO_3$**	0.06 mM	0.5 mM	-
**HCl**	1.1 mM	15.6 mM	8.4 mM
**$CaCl_2(H_2O)_2$**	1.5 mM	0.15 mM	0.6 mM
**α-amilase**	75 U/mL	-	-
**Pepsin**	-	2,000 U/mL	-
**Pancreatin**	-	-	100 U/mL
**Bile salts**	-	-	10 mM

Abbreviations: simulated salivary fluid (SSF), simulated gastric fluid (SGF), simulated intestinal fluid (SIF).

Table S2. Proximal composition of individual (EH-WB, EH-OH, SW and SO) and combined (CI1, CI2 and CI3) ingredients. Values were expressed as g 100 g^{-1} of dry matter.

	Ash	TDF	Fat	Moisture	Proteins	Carbohydrates
**EH-WB**	6,15 ± 0,04 ^{e}	35,29 ± 0,13 ^{d}	3,79 ± 0,11 ^{d}	13,61 ± 0,04 ^{f}	15,52 ± 0,03 ^{f}	60,93 ± 0,06 ^{a}
**EH-OH**	4,28 ± 0,03 ^{d}	89,64 ± 0,10 ^{g}	0,61 ± 0,01 ^{a}	11,07 ± 0,02 ^{d}	3,06 ± 0,07 ^{a}	92,04 ± 0,07 ^{c}
**SW**	2,27 ± 0,34 ^{a}	17,21 ± 1,28 ^{b}	2,17 ± 0,16 ^{b}	11,10 ± 0,03 ^{d}	19,16 ± 0,04 ^{g}	76,40 ± 0,46 ^{b}
**SO**	2,41 ± 0,20 ^{a}	8,81 ± 0,77 ^{a}	9,45 ± 0,75 ^{f}	6,93 ± 0,13 ^{a}	11,52 ± 0,06 ^{c}	76,62 ± 0,89 ^{b}
**CI1**	3,78 ± 0,15 ^{c}	37,74 ± 0,57 ^{e}	4,01 ± 0,26 ^{d}	10,68 ± 0,06 ^{c}	12,32 ± 0,05 ^{d}	76,50 ± 0,37 ^{b}
**CI2**	4,26 ± 0,11 ^{d}	45,98 ± 0,42 f	3,40 ± 0,19 ^{c}	11,23 ± 0,05 ^{e}	11,31 ± 0,05 ^{b}	76,49 ± 0,27 ^{b}
**CI3**	3,30 ± 0,19 ^{b}	29,50 ± 0,72 ^{c}	4,61 ± 0,32 ^{e}	10,12 ± 0,06 ^{b}	13,32 ± 0,05 ^{e}	76,50 ± 0,47 ^{b}

Abbreviations: total dietary fibre (TDF), wheat bran hydrolysate (EH-WB), oat hull hydrolysate (EH-OH), sprouted wheat (SW), sprouted oat (SO), combined ingredient 1, 2 and 3 (CI1, CI2, CI3).

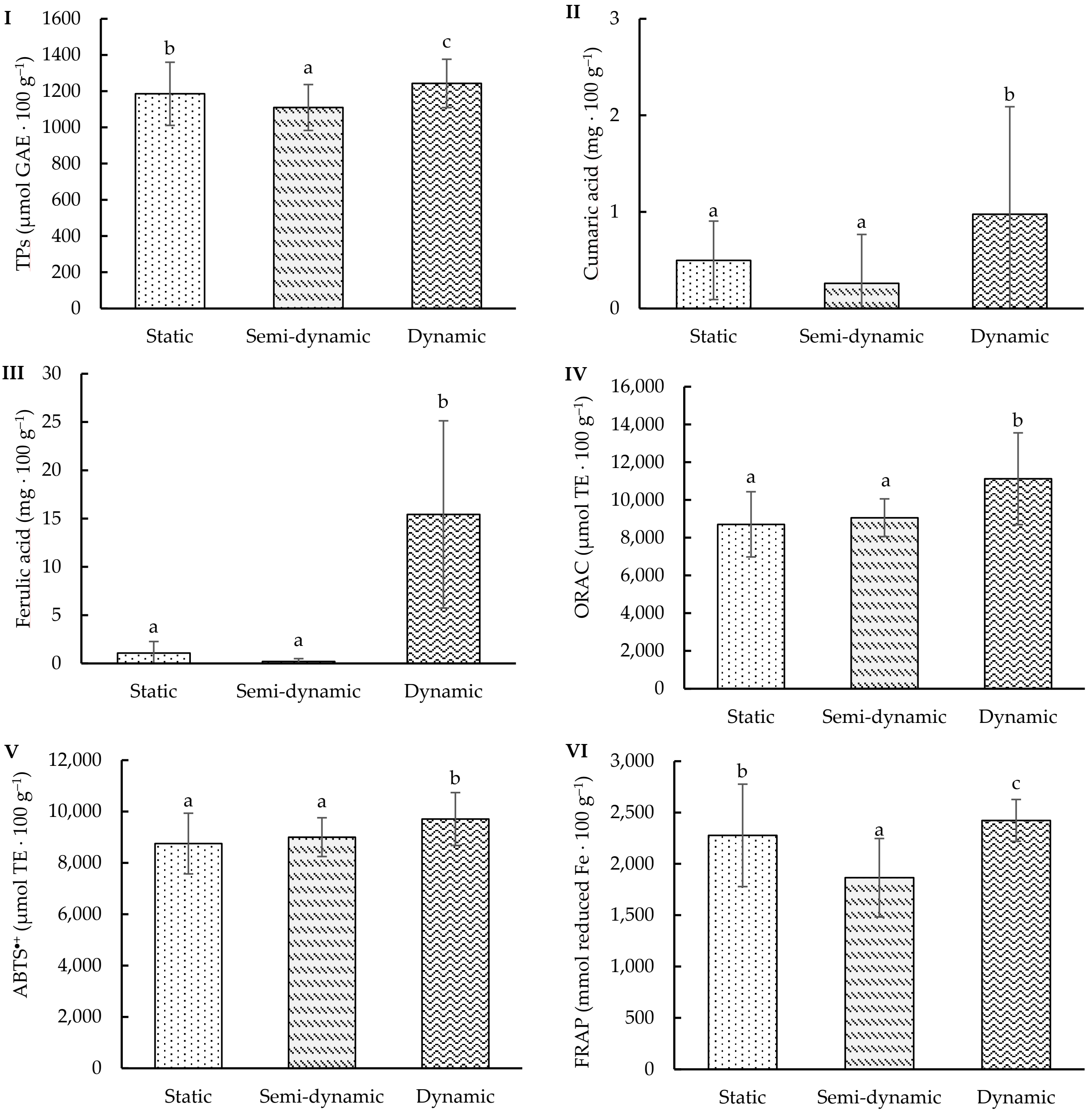

Figure S2. Comparison of the different models of digestion according to the assays of Total Phenolics (TPs, **I**), cumaric acid (**II**), ferulic acid (**III**), ORAC (**IV**), ABTS•+ (**V**) and FRAP (**VI**), Mean values represented as bars, and standard deviations represented as error bars. Different letters indicate significant differences from each other (one-way ANOVA, post hoc Duncan's test, $p < 0.05$).
